# Identification and Characterization of a Multifunctional Biocontrol Agent, *Streptomyces griseorubiginosus* LJS06, Against Cucumber Anthracnose

**DOI:** 10.3389/fmicb.2022.923276

**Published:** 2022-06-02

**Authors:** Chien Hao Chai, Cheng-Fang Hong, Jenn-Wen Huang

**Affiliations:** ^1^Department of Plant Pathology, National Chung Hsing University, Taichung, Taiwan; ^2^Innovative and Development Center of Sustainable Agriculture, National Chung Hsing University, Taichung, Taiwan

**Keywords:** *Streptomyces*, biocontrol, cucumber, anthracnose, *Colletotrichum orbiculare*, plant growth promotion, induced resistance

## Abstract

Twenty-eight bacterial strains isolated from Chinese herb extracts, beer fermentation waste, and raw oyster shells were evaluated for their efficacy in controlling cucumber anthracnose. Four bacterial strains, namely TG01, TG02, LJS06, and LJS08, were found to effectively reduce the mycelial growth of *Colletotrichum orbiculare* COC3 on PDA media. Spraying or drenching LJS06 spore suspension before inoculation significantly *p* < 0.05 reduced disease severity; thus, LJS06 was subject to further characterization. On the basis of the morphological, physiological, and biochemical characteristics and a multilocus sequence analysis of partial 16S rRNA, *atpD*, *rpoB*, and *trpB* genes, LJS06 was identified to be *Streptomyces griseorubiginosus* (Ryabova and Preobrazhenskaya) Pridham et al. Physiological and biochemical tests revealed that *S. griseorubiginosus* LJS06 can produce amylase, cellulase, chitinase, protease, siderophore, polyamines, and indole-3-acetic acid. Thus, a culture filtrate of LJS06 (specifically SL06) was formulated and evaluated for its efficacy against conidial germination, appressorium formation, and anthracnose management. Diluted SL06 was found to significantly (*p* < 0.05) inhibit conidial germination and appressorium formation, which can be attributed to impaired membrane integrity, accumulated reactive oxygen species (ROS), and impaired energy metabolism in the conidia. In addition, the spraying and drenching of diluted SL06 before inoculation consistently and significantly (*p* < 0.05) reduced anthracnose severity. These results jointly suggest that *S. griseorubiginosus* LJS06 can aid in the management of cucumber anthracnose by directly inhibiting conidial function and priming the plant defense system.

## Introduction

Cucumber (*Cucumis sativus* L.) belongs to the Cucurbitaceae family and has a long history of cultivation in Asia (Paris et al., 2012; Jia and Wang, 2021). In Taiwan, its production exceeded 1,800 hectares in 2020, with approximately 88% of the total acreage located in southern and central Taiwan (Council of Agriculture, Executive Yuan, 2020). The average yield of cucumber is more than 25 tons per hectare; from 2015 to 2019, its total market value was approximately US$ 200 million (Council of Agriculture, Executive Yuan, 2020). Most cucumber cultivars are consumed as salad or pickled vegetables, many of which are susceptible to various phytopathogenic fungi during production. Chemical fungicides must thus be applied (Meera et al., 1994; Shoresh et al., 2005; El-Tarabily et al., 2009), particularly in hot and humid environments such as southern and central Taiwan.

*Colletotrichum orbiculare* (syn. *C. lagenarium*) infection of cucumber leaves, stems, or fruits can result in severe yield and quality loss (Kwack et al., 2005). Currently, only a few fungicides are registered for managing cucurbit anthracnose in Taiwan. Many of these are demethylation inhibitors (DMIs) or quinone outside inhibitor (QoI) fungicides. Excessive fungicide use poses threats to the environment and may promote fungicide resistance (Shimizu et al., 2013). Therefore, finding effective fungicide alternatives is crucial not only to maintain cucumber production but also to fulfill consumers’ expectation of food safety.

The use of beneficial microorganisms for managing crop diseases has received considerable attention because of their versatile capabilities in acting as biocontrol agents. Actinomycetes, particularly *Streptomyces* spp., constitute a large part of the rhizosphere microbiome (Shih et al., 2003; Olanrewaju and Babalola, 2019). Many studies have used *Streptomyces* to manage airborne or soil-borne plant diseases, demonstrating that *Streptomyces* can produce various antimicrobial compounds or enzymes or act as a hyperparasite, thereby inhibiting plant pathogens and diseases (Köhl et al., 2019; Olanrewaju and Babalola, 2019). For example, Palaniyandi et al. (2013) reported that *S. phaeopurpureus* ExPro138 can produce several proteases, inhibiting spore germination and appressorium formation, act as a hyperparasite of *Colletotrichum coccode*s, and reduce the incidence of tomato anthracnose (Palaniyandi et al., 2013). Shih et al. (2003) and Yang et al. (2021) reported that the fungichromin produced by *S. padanus* PMS-702 can be used to control *Rhizoctonia* damping-off on cabbage, sheath blight on rice, *Phytophthora* fruit rot on peach, and downy mildew on cabbage (Shih et al., 2003; Yang et al., 2021).

In addition to providing direct inhibition, *Streptomyces* species can promote plant growth and induce plant resistance to multiple diseases (Pieterse et al., 2014; Vurukonda et al., 2018; Abbasi et al., 2019; Mun et al., 2020). Mun et al. (2020) reported that cucumber plants drenched with *Streptomyces* sp. LH4 spore suspension were significantly taller and wider than plants that were not. Abbasi et al. (2019) reported that the dry weight and fresh weight of tomato plants increased by 29–36% and 22–37%, respectively, after 60 days of drenching with the spore suspension of *S. enissocaesilis* IC10 and *S. rochei* Y28 in potting soil. The peroxidase activity of tomato plants treated with *S. rochei* Y28 was also increased (Abbasi et al., 2019). *Streptomyces* species have also been isolated from the rhizosphere and healthy plant tissues (Mundt and Hinkle, 1976; Sardi et al., 1992; Sturz and Nowak, 2000), indicating that *Streptomyces* are symbiotic species, which may acquire nutrients from their host plants and protect them from various pathogens (Vurukonda et al., 2018). These findings demonstrate the capabilities of *Streptomyces* species as beneficial microbes in agriculture.

Food safety and environmental conservation have become increasingly important to the general public. Maintaining yield and quality with minimum input while satisfying consumer expectations and the requirement for minimal residue during a long harvest period is highly challenging, thereby necessitating the development of fungicide alternatives and disease management strategies in conventional and organic farming. Because cucumber is an essential produce for raw consumption and processed vegetables in Taiwan, in the present study, we (1) screened for multifunctional bacterial strains for managing cucumber anthracnose; (2) identified the antagonistic microorganism, characterizing the biological features of the selected antagonist; and (3) elucidated the potential mechanisms underlying *C*. *orbiculare* inhibition and anthracnose severity reduction.

## Materials and Methods

### Bacterial Strains and Phytopathogenic Fungus

The bacterial strains assessed in this study were isolated from the soil of a cucumber farm (24°12′35.0”N 120°31′43.9”E), Chinese herb extracts, beer fermentation waste, and raw oyster shells. To isolate potential biocontrol bacteria, 10 g of soil sample, Chinese herb extract, beer fermentation waste, and oyster shell powder (obtained by crushing raw oyster shells with a hammer) were suspended individually in 100 mL of sterile distilled water and serially diluted, and 0.1 mL of each diluted suspension was spread on humic acid–vitamin agar (Hayakawa and Honomura, 1987) and nutrient agar (NA, Difco™, MI, United States). The bacterial colonies obtained were purified on International *Streptomyces* Project (ISP)-4 medium (Difco™, MD, United States) and NA plates for further experiments.

A fungal pathogen causing cucumber anthracnose was isolated from a symptomatic cucumber leaf in Dali District, Taichung City. On the basis of the morphological characteristics and the internal transcribed spacer sequences (Supplementary Table 1; White et al., 1990; Gardes and Bruns, 1993), the cucumber anthracnose pathogen was identified to be *C. orbiculare* COC3 (Chai, unpublished data). Koch’s postulate was satisfied, thus demonstrating the pathogenicity of the isolate. The fungal isolate is currently long-term stored in the Department of Plant Pathology, National Chung Hsing University.

### Antagonistic Activity of Bacterial Strains Against *Colletotrichum orbiculare*

To evaluate the antifungal activity of bacterial strains, mycelial plugs (8 mm in diameter) of a 9-day-old *C. orbiculare* COC3 were taken from the margin of a colony on potato dextrose agar (PDA, Difco™, United States). The mycelial plugs were placed on the center of potato nutrient agar (PNA) media (19.5 g of PDA and 7.5 g of NA in 1 L of water) in 8.5-cm Petri dishes. Subsequently, a filter paper disk (80 mm in diameter) containing 20 μL suspension of each bacterial strain was placed 2.5 cm from the mycelial plug of *C. orbiculare* COC3. A filter paper disk with sterile distilled water was placed on the other side, 2.5 cm from the mycelia disk, as an untreated control. Each bacterial strain had three dishes. The dishes were incubated at room temperature for 4–7 days, and the length of mycelium on treated and untreated sides were measured. The mean antagonistic activity of three replications of each bacterial strain was calculated using the formula: antagonistic activity = [(mycelial length on the untreated side – mycelial length on the treated side)/mycelial length on the untreated side] × 100. Bacterial strains that were highly antagonistic were selected for further experiments.

### Effect of Antagonistic Bacterial Strains on Growth Promotion and Anthracnose Severity of Cucumber

To determine the effect of the antagonistic bacterial strains in promoting plant growth, 10 mL of the spore suspension (1 × 10^8^ CFU/mL) of each bacteria strain TG01, TG02, LJS06, and LJS08 was drenched weekly in the soil of 7-day-old cucumber seedlings for 3 consecutive weeks (Patel et al., 2018). Cucumber plants treated with sterile distilled water were included as blank controls. The plants were incubated at 25°C with 12 h of photoperiod. The plant height and the root length were measured when the plants were 28 days old. All treatments were conducted with five replications, and the experiment was conducted twice.

To determine the efficacy of the bacterial strains against cucumber anthracnose, the spore suspension of each bacterial strain was drenched in the soil of cucumber plants following the aforementioned method. One day after the last drench were the cucumber plants spray inoculated with 10 mL of *C. orbiculare* COC3 conidial suspension (1 × 10^5^ spores/mL) and incubated at 25°C with a 12-h photoperiod. The disease severity (the percentage of diseased area on the leaf) of the first four true leaves from the cotyledon of inoculated plants was evaluated 11 days after inoculation. There were four plants in each treatment, and the experiments were conducted twice.

### Culture, Physiological, and Biochemical Characteristics of the *Streptomyces* Strains

The morphology of the colony, color of aerial and substrate mycelia, and soluble pigment of strains TG01, TG02, LJS06, and LJS08 were visually estimated using the ISCC-NBS color system (Kelly, 1964; Centore, 2016) after 7 days of incubation on ISP-2, ISP-3, ISP-4, and ISP-5 media, respectively (Shirling and Gottlieb, 1966). The capability of utilizing different carbon sources for each bacterial strain was evaluated on ISP-9 medium amended with 1.0% of L-arabinose, cellulose, D-fructose, D-glucose, *l*-inositol, D-mannitol, raffinose, sucrose, or D-xylose and incubated at 28°C for 14 days. However, melanin production was evaluated on ISP-1 and ISP-7 media, and hydrogen sulfide production was evaluated on ISP-6 medium after 2 days of incubation at 28°C (Shirling and Gottlieb, 1966; Lechevalier, 1980).

To observe the spore production and spore surface ornamentation of the bacterial strain LJS06, mycelial disks were taken from the colony on ISP-3 after 7 days of incubation and fixed in 0.1 M phosphate buffer diluted glutaraldehyde solution (2.5%) at 4°C for 16 h. Fixed disks were washed with 0.1 M phosphate buffer, dehydrated in 25, 50, 60, 70, 80, 90, and 95% ethanol, and dried in a critical point dryer. The specimens were then mounted on a sputter coater, coated with a thin layer of gold-palladium alloy for 60 s, and observed under a scanning electron microscope (JEOL JSM-7800F, Japan).

### Enzymatic Activity and Capability of Producing Siderophore, Polyamines, and Indole Acetic Acid

The production of amylase, cellulases, chitinase, and proteolytic enzymes was determined following the methods described by previous studies (Khokhar et al., 2011; Halimahtussadiyah et al., 2017; Perwendha et al., 2020). In each medium for testing enzymatic activity, a clear zone surrounding the colony indicated the production of enzymes. The enzymatic index was expressed by the relationship between the average diameter of the clear zone and average diameter of the colony growth. The enzyme activity index (EI) was calculated using the following formula:


EI=(diameter⁢of⁢clear⁢zone-diameter⁢of⁢colony)/diameter⁢of⁢colony.

Enzymatic activity is considered strong if EI > 1 (Duncan et al., 2006; Lechuga et al., 2016; Ntabo et al., 2018). There were five plates for each enzyme test, and the whole experiment was conducted twice.

Siderophore production was determined using the chrome azurol S (CAS) assay (Schwyn and Neilands, 1987). The four bacterial strains TG01, TG02, LJS06, and LJS08 were spot inoculated on CAS plates and incubated at 28°C for 4 days. The development of an orangish-yellow halo around the colony indicates siderophore production. There were five plates for each bacterial strain, and the whole experiment was conducted twice.

To test the capability of producing arginine decarboxylase, the four bacterial strains were cultured in a modified decarboxylase medium amended with 2 g L^–1^ of L-arginine and 0.01 g L^–1^ of bromocresol purple as a pH indicator. The decarboxylating strain was detected when the bacterial colony was surrounded by a deep purple halo (Nassar et al., 2003).

To evaluate the production of indole-3-acetic acid (IAA), each strain was cultured in Luria Bertani broth (LB, Difco™, United States) containing 5 mM L-tryptophan at 30°C for 5 days. One milliliter of the cultural broth of each bacterial strain was sampled every day and mixed with 2 mL of Salkowski reagent (0.5 M FeCl_3_: 98% H_2_SO_4_: ddH_2_O = 1:30:50). The appearance of a pink color in the reagent indicated IAA production, which was then quantified at OD 530 (Glickmann and Dessaux, 1995). Each bacterial strain had five replicates, and the whole experiment was conducted twice.

### Molecular Identification of the *Streptomyces* Strains

To identify the bacterial strains, spores of each strain were harvested by gently scratching the 7-day-old colony on ISP-2 using a sterile inoculum loop. The spores were suspended in a 1.5-mL centrifuge tube containing 1 mL of 12.5 mM NaOH. The mixture was incubated at 98°C for 15 min and centrifuged at 2,000×*g* for 1 min, and the supernatant was used as a DNA template for PCRs. The partial sequence of the 16S rRNA of each bacterial strain was Polymerase chain reaction (PCR) amplified using St-F and St-R primers (Supplementary Table 1). Each PCR had 1 μL of each primer, 5 μL of PCR master Mix Kit, and 1 μL of template DNA, and 2 μL of ddH_2_O was added for a final volume of 10 μL. PCR was conducted under the following thermocycler conditions: initial denaturation at 95°C for 2 min; subsequent sets of 35 cycles of 95°C for 1 min, 57°C for 1 min, and 72°C for 1 min; and a final extension at 72°C for 15 min.

To perform a multilocus sequence analysis (MLSA) for the *Streptomyces* sp. strain LJS06, additional housekeeping genes, including *atpD* (ATP synthase F1, β-subunit), *rpoB* (RNA polymerase, β-subunit), and *trpB* (tryptophan synthase, β-subunit), were amplified with the primers used in Guo et al. (2008) (Supplementary Table 1). PCR was performed per the aforementioned procedure under the following thermocycler conditions: initial denaturation at 95°C for 2 min; subsequent sets of 35 cycles of 95°C for 1 min, 65°C for 1 min, and 72°C for 2 min; and a final extension at 72°C for 15 min.

The PCR products of the 16S rRNA and the three housekeeping genes were sequenced using an Applied Biosystem 3730 DNA analyzer at Tri-Biotech (Taipei, Taiwan). The quality of the obtained sequences was assessed using Geneious Prime v. 2021.2.2, blasted and aligned with reference sequences, and deposited in NCBI GenBank (Supplementary Table 2). For the 16S rRNA, a phylogenetic tree was constructed using the Maximum Likelihood method with the Tamura-3-parameter model and 1,000 bootstrap replicates. The three housekeeping genes were combined, and a phylogenetic tree was constructed using the Neighbor Joining method with the Tamura–Nei model and 1,000 bootstrap replicates. Both phylogenetic trees were constructed using MEGA X (Nei and Kumar, 2000; Kumar et al., 2018).

### Effect of *Streptomyces griseorubiginosus* LJS06 Culture Filtrate on the Germination and Appressorium Formation of *Colletotrichum orbiculare* and Its Potential Mechanisms

*S. griseorubiginosus* LJS06 was cultured in a soybean meal broth [SMG, 1% glucose (w/v), 0.5% soybean meal (w/v), 1 L distilled water] for 5 days, and the effect of the culture filtrate, namely SL06, on the conidial germination and appressoria formation of *C*. *orbiculare* was evaluated. To test the conidial germination and appressoria formation, 10 μL of 100-fold-diluted SL06 was mixed with 10 μL of *C*. *orbiculare* conidial suspension (1 × 10^4^ spores/mL), resulting in 200× dilution of SL06 in each droplet of the mixture on glass slides. Conidial suspension treated with sterile water and SMG broth were included as negative controls. The mixtures were covered with coverslips, and the slides were placed on V-shaped glass rods in moist chambers and incubated at 25 ± 2°C. The percentages of conidial germination and appressorium formation of approximately 100 conidia were recorded 24 h after incubation. Each treatment had four replicates, and the experiment was conducted three times.

#### Membrane Integrity

The effect of SL06 on the membrane integrity of *C. orbiculare* COC3 was evaluated using a method described by previous studies (Chen and Dickman, 2005; Tang et al., 2014). Briefly, 15 μL of 100-fold-diluted SL06 was mixed with an equal volume of the conidial suspensions, resulting in a 200-fold dilution of the mixture. Next, 20 μL of the mixture was incubated in a concave slide and maintained in a moist chamber at 25°C for 24 h. Treatments with sterile water and SMG broth were included as negative controls. The treated conidia were stained with 10 μL of 0.05% Evans blue for 45 min before observation. Conidia uptook the stain and turned blue, suggesting that membrane integrity was impaired.

#### Accumulation of Reactive Oxygen Species

To evaluate the oxidative stress in *C. orbiculare* COC3 conidia after treatment with SL06 (200-fold dilution), the conidia were stained with dihydrorhodamine 123 (DHR 123) for 30 min and observed under a fluorescent microscope with an optical filter set XF104-2 (Leica, Germany). Conidia treated with sterile water and SMG broth were included as negative controls. Oxidation of the non-fluorescent DHR 123 into green fluorescent Rhodamine 123 (Rh123) under the fluorescent microscope suggested accumulation of reactive oxygen species (ROS) in the conidia (Kannan and Jain, 2000).

#### Interference of Energy Metabolism

To determine whether the energy metabolism of *C. orbiculare* COC3 was interfered with after treatment with SL06, an equal volume of 100-fold-diluted SL06 and the conidial suspension were mixed. Pyraclostrobin (23.6% emulsion, 3,000-fold dilution), a fungicide known to block the electron transfer at the quinone outside site of the bc1 complex, thereby inhibiting the energy metabolism of the pathogen, was included as a positive control, and SMG broth was included as a negative control. Subsequently, 180 μL of each mixture was loaded into a 96-well microplate (Costar, United States) and incubated in the dark at 25°C for 24 h before staining with Alamar blue (Life Technologies, CA, United States). The relative fluorescent unit in each treatment was determined using a Tecan Infinite M200 plate reader with absorbances at 570 and 600 nm (Pettit et al., 2005; Cox et al., 2009; Sharma et al., 2019). The color change from non-fluorescent blue to highly fluorescent pink indicated normal respiration in *C. orbiculare* COC3 conidia and was photographed using a digital camera.

### Effect of *Streptomyces griseorubiginosus* LJS06 Culture Filtrate on Anthracnose Severity of Cucumber

To test the effect of application methods and different dilutions of SL06 on anthracnose severity, 10 mL of 20-, 100-, and 200-fold diluted SL06 were either sprayed on the leaves or drenched in the soil of cucumber plants with four unfolded leaves, respectively (Fan et al., 2019). Cucumber plants treated with sterile distilled water and diluted SMG broth were included as negative controls. After 24 h of each treatment, the entire cucumber plant was spray inoculated with conidial suspension (1 × 10^5^ spores/mL) of *C. orbiculare* COC3. The disease severity of anthracnose in each treatment was assessed per the aforementioned method. Each treatment had six plants, and the entire experiment was conducted twice.

### Statistical Analysis

All experiments in this study were performed at least twice. The data are presented in terms of the mean and standard error of the values obtained from at least three replicates. The treatment effects were determined using a one-way analysis of variance at *p* = 0.05. Multiple comparisons were performed using Fisher’s least significant difference test at *p* = 0.05 in SAS Enterprise Guide 6.1 (SAS Institute, Cary, NC, United States).

## Results

### Screening for Multifunctional Antagonistic Bacteria Against Cucumber Anthracnose

Twenty-eight bacterial strains were obtained from various materials. Five isolates, namely TG01, TG02, LJS05, LJS06, and LJS08, were found to effectively inhibit more than 40% of the mycelial growth of *C. orbiculare* COC3 on PDA. Eleven strains exhibited 26–40% of inhibition, whereas the remaining strains had <25% inhibition (Table 1). On the basis of the *in vitro* antagonistic activity, four *Streptomyces* strains—TG01, TG02, LJS06, and LJS08—were selected. The plant growth promotion properties and disease inhibition capability of the four bacterial strains were further evaluated.

**TABLE 1:** Efficacy of bacterial strains on inhibiting the mycelial growth of *Colletotrichum orbiculare* COC3.

Bacterial isolates	Mycelial inhibition^a^
LJS06	>60%
TG01, TG02, LJS05, LJS08	41–60%
LJS02, LJS03, LJS04, LJS07, TG05, TG07, WB01, HT1, CD1-2, CD1-4, CD4	26–40%
LJS01, TG06, CD1-3	11–25%
HT3	0–10%
TG03, TG04, WB02, WB03, HT2, CD1-1, CD2, CD3	<0%

*^a^Mycelial inhibition (%) = [(colony radius in control–colony radius in treatment)/(colony radius in control)] × 100.*

In the experiment on plant growth promotion, spore suspensions of TG01, TG02, LJS06, and LJS08 were drenched in the soil of 7-day-old cucumber plants for 3 consecutive weeks. Among these, the LJS06-treated plants had the greatest plant height after 28 days in both trials. Although the growth promotion effect of LJS06 was not significant compared with the untreated control in the first trial, LJS06-treated plants were significantly (*p* < 0.05) taller than those treated with other strains (Table 2). However, TG01 consistently and significantly (*p* < 0.05) enhanced the root growth of cucumbers, followed by LJS06 and TG02 (Table 2).

**TABLE 2:** Effect of spore suspension of bacterial strains TG01, TG02, LJS06, and LJS08 on plant height and root length of cucumber seedlings.

Treatment^a^	Plant height (cm)^b^				Root length (cm)			
	Experiment 1		Experiment 2		Experiment 1		Experiment 2	
TG01	22.9 ± 0.5	c^d^	30.8 ± 1.0	b	20.1 ± 1.8	a	49.4 ± 1.1	a
TG02	24.5 ± 0.7	bc	27.8 ± 0.6	c	19.7 ± 1.9	a	26.6 ± 0.8	bc
LJS06	29.8 ± 0.6	a	35.5 ± 0.3	a	14.5 ± 0.7	bc	27.6 ± 0.6	b
LJS08	22.8 ± 1.1	c	21.5 ± 0.2	e	19.6 ± 0.2	ab	23.2 ± 1.1	d
sdH_2_O^c^	27.5 ± 1.9	ab	25.8 ± 0.7	d	14.5 ± 0.3	c	24.8 ± 0.9	cd

*^a^Ten milliliters of spore suspension (1 × 10^8^ CFU/mL) of each antagonistic bacterial strain was drenched weekly in the soil of 7-day-old seedlings for 3 consecutive weeks.*

*^b^Plant height and root length were estimated when the seedlings were 28 days old.*

*^c^Plants drenched with sterile distilled water were included as untreated control.*

*^d^Means (n = 5) (± standard error) in the same column followed by different letters are significantly different (p < 0.05) according to Fisher’s least significant difference test.*

**FIGURE 1:**
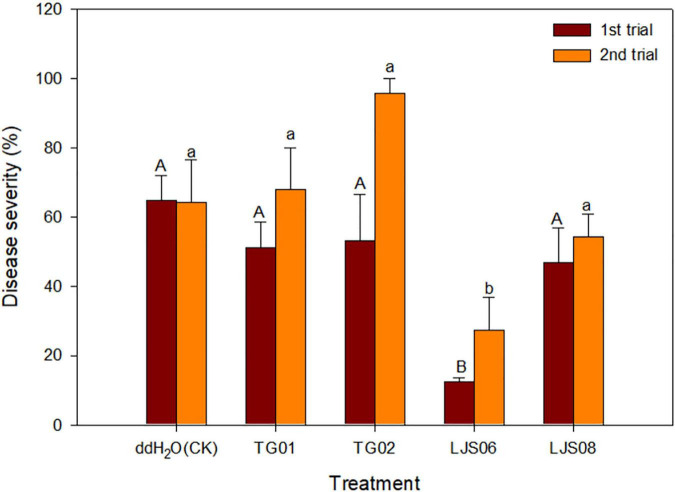
Effect of spore suspension of four antagonistic bacterial strains on disease severity of cucumber anthracnose caused by *Colletotrichum orbiculare* COC3. Means (*n* = 4) in the same trial indicated by different letters significantly differ (*p* < 0.05) according to Fisher’s least significant difference test. Bar = standard error.

**FIGURE 2:**
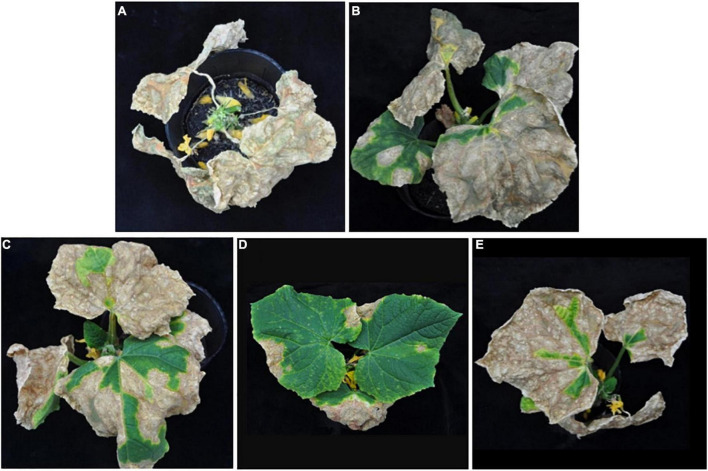
Effect of antagonistic bacterial strains on anthracnose severity of cucumber seedlings 11 days after inoculation. Only one representative plant is shown in each treatment drenched with **(A)** distilled water as the control, **(B)** strain TG01, **(C)** strain TG02, **(D)** strain LJS06, and **(E)** strain LJS08.

*In planta* experiments indicated that LJS06 significantly (*p* < 0.05) reduced the average disease severity of cucumber anthracnose 11 days after inoculation. By contrast, drenching the spore suspension of TG01, TG02, and LJS08 only slightly decreased the disease severity in the first trial. However, the disease management effect for the three strains was inconsistent in the second trial (Figures 1, 2).

### Culture Characteristics, Scanning Electron Microscope Morphology, and Multilocus Sequence Analysis of LJS06

The four tested bacterial strains grew well on all the tested ISP media (Table 3). For LJS06, the aerial mycelia were light gray to reddish gray, which turned into purplish white on ISP-5 medium after 7 days of incubation. The substrate mycelials were light yellow to yellowish brown, but no pigmentation was observed in any ISP medium (Table 3 and Supplementary Figure 1). Scanning electron microscopy (SEM) revealed that LJS06 had a smooth spore surface; the spore chains were straight or flexuous, with more than 20 spores per chain, indicating rectiflexible type of spore production (Supplementary Figure 3). In the carbon source utilization test, LJS06 used all the tested carbon sources, whereas the other strains only used a limited number of carbon sources (Table 4). In the melanin and H_2_S production tests, LJS06 produced both compounds, whereas the capability of producing melanin or H_2_S was variable in the other strains (Table 4).

**TABLE 3:** Culture characteristics of the four *Streptomyces* strains on ISP-2, ISP-3, ISP-4, and ISP-5 media.

Strain	Culture characteristics in International *Streptomyces* Project Medium (ISP) media^a^											
	ISP-2			ISP-3			ISP-4			ISP-5		
	AMC^b^	SMC	SP	AMC	SMC	SP	AMC	SMC	SP	AMC	SMC	SP
TG01	Light gray^c^	Deep yellow	Y^d^	Light brownish gray	Strong brown	B	Light gray	Light greenish yellow	N	Light gray	Pale greenish yellow	N
TG02	Light gray	Deep yellow	Y	Brownish gray	Dark brown	B	Light gray	Light greenish yellow	N	Yellowish gray	Pale greenish yellow	N
LJS06	Reddish gray	Vivid yellow	N	Light gray	Dark yellowish brown	N	Light gray	Pale yellow	N	Purplish white	Light yellow	N
LJS08	Grayish red	Vivid reddish orange	N	Grayish pink	Vivid pink	R	Grayish red	Strong pink	N	Pinkish white	Light orange	N

*^a^ISP-2: yeast extract-malt agar; ISP-3: oatmeal agar; ISP-4: inorganic starch agar; ISP-5: glycerol asparagine agar.*

*^b^AMC, aerial mycelium color; SMC, substrate mycelium color; SP, soluble pigment.*

*^c^Colors were determined based on the ISCC–NBS color charts.*

*^d^N, no pigment; B, brown pigment; R, red pigment; Y, yellow pigment.*

**TABLE 4:** Capability for carbon utilization and melanin and H_2_S production for the four *Streptomyces* strains.

Strain	ISP-1^a^	ISP-6^b^	ISP-7^a^	Carbon utilization^c^								
				Ara	Cel	Fru	Glu	Ino	Man	Raf	Suc	Xyl
TG01	–^d^	+	–	–	–	+/–	+	–	–	–	–	+/–
TG02	–	+	–	–	–	+/–	+	–	–	–	–	+/–
LJS06	+	+	+	+	+	+	+	+	+	+	+	+/–
LJS08	–	–	–	+/–	–	+/–	+	+/–	+	–	+/–	+/–

*^a^ISP-1 and ISP-7: For detecting melanin production.*

*^b^ISP-6: For detecting H_2_S production.*

*^c^ISP-9 medium plates were amended with 1% (w/v) of different carbon sources: Ara, L-arabinose; Cel, cellulose; Fru, D-fructose; Glu, D-glucose; Ino, l-inositol; Man, D-mannitol; Raf, raffinose; Suc, sucrose; Xyl, D-xylose.*

*^d^+, Positive reaction; –, Negative reaction; +/−, Indeterminate reaction.*

**FIGURE 3:**
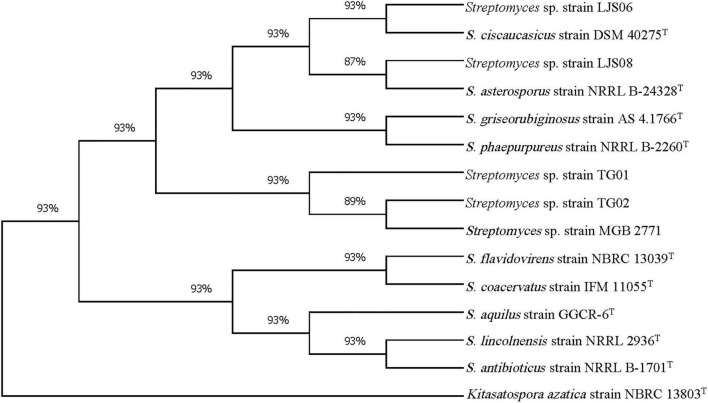
Phylogenetic tree of *Streptomyces* strains TG01, TG02, LJS06, and LJS08 based on 16S rRNA sequences. The tree was constructed using the maximum likelihood method with Tamura-3- parameter model and 1,000 bootstrap replicates in MEGA X. *Kitasatospora azatica* strain NBRC 13803^T^ was used as an outgroup.

**FIGURE 4:**
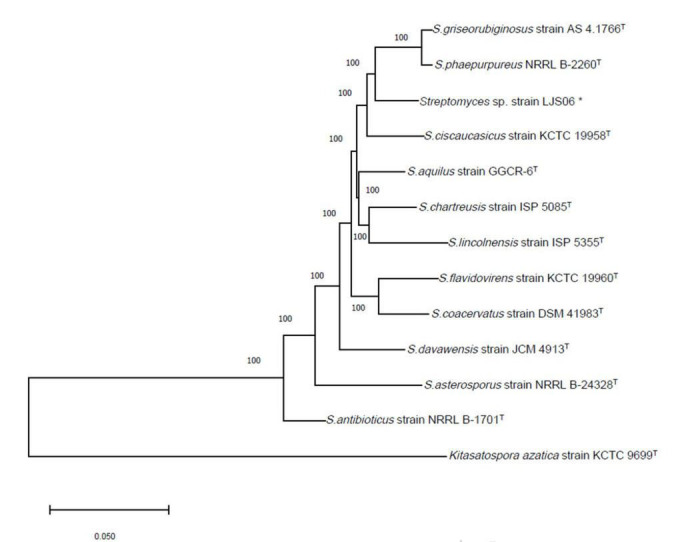
Phylogenetic tree of *Streptomyces* species based on concatenated sequences of partial *atpD*, *rpoB*, and *trpB* genes. The tree was constructed using the Neighbor Joining method with the Tamura-Nei model and 1,000 bootstrap replicates in MEGA X. *Kitasatospora azatica* strain KCTC 9699^T^ was used as an outgroup. *Indicates the strain used in this study.

PCR amplification of the 16S rRNA gene generated approximately a 1,000-bp fragment from the four bacterial strains. The 16S rRNA gene sequences were BLASTed against the reference sequences (Accession No. MN339842, AY508512, and AY999902) from the NCBI GenBank. All genes had a more than 96% similarity with the reference strains in *Streptomyces*. On the basis of the 16S rRNA sequences, both strains TG01 and TG02 were identified as *Streptomyces* sp. (MN339842), whereas, strains LJS06 and LJS08 were identified as *S. ciscaucasicus* (AY508512) and *S. asterosporus* (AY999902), respectively (Figure 3). Further identification of LJS06 based on MLSA revealed that LJS06 was clustered with *S. griseorubiginosus* strain AS 4.1766^T^ and *S. phaepurpureus* strain NRRL-B 2260^T^ in the phylogenetic tree (Figure 4). On the basis of the culture, physiological, biochemical characteristics, SEM morphology, and MLSA results, LJS06 was identified to be *S. griseorubiginosus* (Ryabova and Preobrazhenskaya) Pridham et al. The 16S rRNA, *atpD, rpoB*, and *trpB* sequences of the *S. griseorubiginosus* strain LJS06 were deposited in GenBank under the accession numbers OK668212, OL322103, OL322104, and OL322105, respectively (Supplementary Table 2).

### *Streptomyces griseorubiginosus* LJS06 Can Produce Hydrolytic Enzymes, Siderophore, Polyamines, and Indole-3-Acetic Acid

The four *Streptomyces* strains were tested for their capability to produce hydrolytic enzymes, siderophores, polyamines, and IAA in media. In the enzyme production experiments, LJS06 had the highest EI among the tested *Streptomyces* strains, suggesting a good capability for producing amylase, cellulase, chitinase, and protease (Table 5). Of the four strains, only LJS06, TG01, and TG02 turned the greenish-blue CAS media into yellow, indicating siderophore production capability (Table 5 and Supplementary Figure 2). In addition, the four strains exhibited deep purple halos around and beneath the colonies in modified decarboxylase media, suggesting polyamine production capability. Although all tested strains could also produce IAA, the quantity produced by LJS06 was significantly higher than that by the other strains after 4 days of incubation (Table 6).

**TABLE 5:** Enzyme activity indexes and polyamine and siderophore production by the four *Streptomyces* strains.

Strain	Enzyme Activity Index (EI)^a^					
	Amylase	Cellulase	Chitinase	Protease	Polyamines	Siderophore (mm)
TG01	1.41	0.73	0.15	1.23	++^b^	14.0 ± 0.3^c^
TG02	1.50	0.68	0.11	0.89	++	14.0 ± 0.5
LJS06	2.57	1.65	1.11	1.77	++	15.7 ± 0.4
LJS08	0.13	0	0	0.69	++	NA^d^

*^a^Enzyme activity index (EI) = (Halo zone diameter–Colony diameter)/Colony diameter.*

*^b^+: radius < 30 mm; ++: radius ≥ 30 mm.*

*^c^Means (n = 4) (± standard error) of the yellow halo diameter on CAS media.*

*^d^No yellow halo was formed.*

**TABLE 6:** Production of indole-3-acetic acid by four *Streptomyces* spp. in LB broth containing 5 mM of l-tryptophan at 30°C for 5 days.

Strain	IAA concentration (mg/L)									
	1st day		2nd day		3rd day		4th day		5th day	
TG01	17.9 ± 3.9	A*^a^*	23.3 ± 3.9	B	21.8 ± 2.2	B	22.3 ± 0.4	C	22.6 ± 2.3	C
TG02	25.5 ± 3.5	A	53.4 ± 3.0	A	51.5 ± 1.7	A	51.2 ± 3.0	B	45.8 ± 3.1	B
LJS06	2.8 ± 0.4	B	57.0 ± 0.2	A	59.2 ± 4.2	A	62.4 ± 1.9	A	57.6 ± 3.1	A
LJS08	0.8 ± 0.1	B	10.0 ± 0.4	C	10.6 ± 0.4	C	9.5 ± 0.2	D	7.8 ± 1.3	D

*^a^Means (n = 5) (± standard error) in the same column followed by different letters are significantly different (p < 0.05) according to Fisher’s least significant difference test.*

### LJS06 Culture Filtrate Inhibited Conidial Germination and Appressorium Formation by Impairing Membrane Integrity, Inducing Reactive Oxygen Species Accumulation, and Impairing Energy Metabolism in *Colletotrichum orbiculare*

**FIGURE 5:**
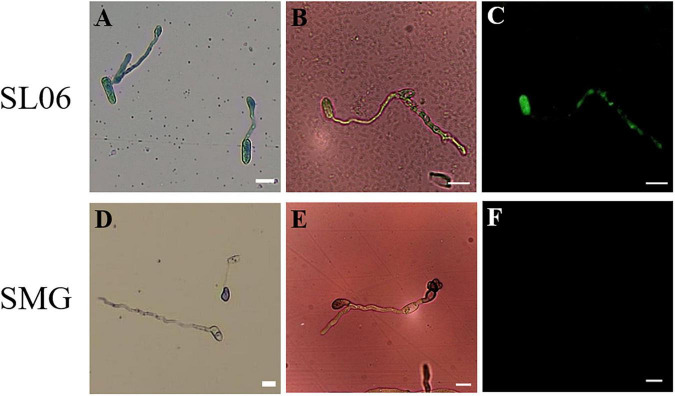
Effect of *Streptomyces griseorubiginosus* LJS06 culture filtrate (SL06) on membrane integrity and oxidative status of the conidia of *Colletotrichum orbiculare*. The conidia treated with diluted SL06 were stained with Evans blue, suggesting impaired membrane integrity of the conidia **(A)**. Additionally, green fluorescence was observed in SL06-treated conidia, suggesting reactive oxygen species accumulation in the conidia **(B,C)**. By contrast, SMG broth-treated conidia was not stained with Evans blue **(D)**, and no fluorescence was observed, suggesting intact cell membrane and normal oxidative status in the conidia **(E,F)**. Bar = 10 μm.

**FIGURE 6:**
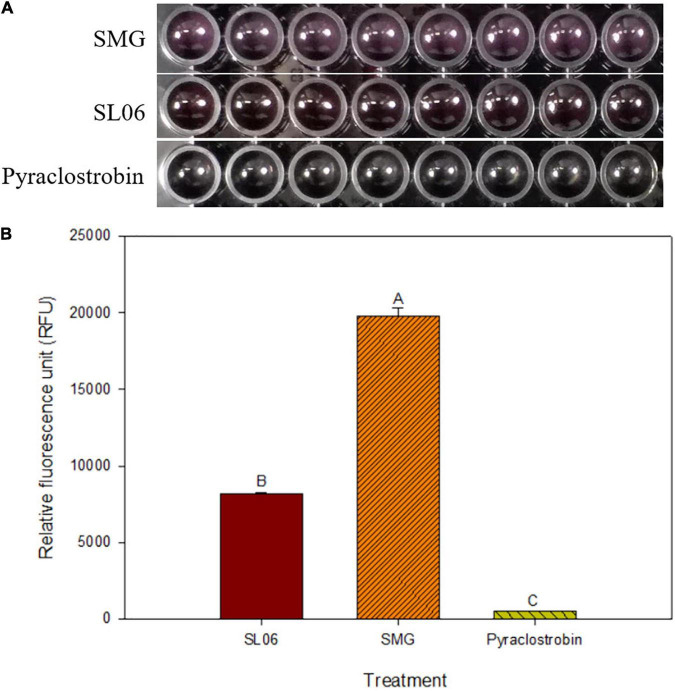
Effect of *Streptomyces griseorubiginosus* LJS06 culture filtrate (SL06) on the energy metabolism of *Colletotrichum orbiculare* COC3. **(A)** Alamar blue turned pink in soybean meal broth-treated conidial suspension but remained unchanged in SL06-treated and pyraclostrobin-treated conidial suspensions after 24 h. **(B)** SMG-treated conidial suspension had significantly higher relative fluorescence units than SL06 and pyraclostrobin treatments, suggesting that the energy metabolism was impaired in the latter treatments. Means (*n* = 8) followed by different letters are significantly different (*p* < 0.05) according to Fisher’s least significant difference test. Bar = standard error.

The efficacy of 200-fold-diluted SL06 on inhibiting the conidial germination and appressorium formation of *C. orbiculare* was evaluated. Compared with the SMG broth and water controls, diluted SL06 significantly (*p* < 0.05) reduced the conidial germination and appressorium formation after 24 h (Table 7). To investigate the potential mechanism underlying direct inhibition, the diluted SL06–treated conidia were stained with Evans blue and examined under a compound microscope. After 24 h, the diluted SL06–treated conidia were permeable to Evans blue, whereas SMG broth–treated conidia were not, suggesting that the membrane integrity of *C. orbiculare* COC3 was impaired by the metabolites (Figures 5A,D). *C. orbiculare* COC3 conidia were also stained with DHR123 to evaluate whether the metabolites in diluted SL06 could change the oxidative status in the conidia. Green fluorescence was observed in diluted SL06–treated conidia but not in SMG broth–treated conidia, suggesting that the metabolites in diluted SL06 may induce ROS accumulation, thereby increasing oxidative stress in the conidia of *C. orbiculare* (Figures 5B,C,E,F). Moreover, conidial suspension treated with either pyraclostrobin or diluted SL06 remained blue to deep purple, suggesting the inhibition of energy metabolism in the conidia. Conversely, SMG broth–treated conidia reduced Alamar blue into highly fluorescent pink resorufin, thereby exhibiting the highest relative fluorescence unit (*p* < 0.05) among all treatments (Figure 6).

**TABLE 7:** Effect of *Streptomyces griseorubiginosus* LJS06 culture filtrate (SL06) on conidial germination and appressorium formation of *Colletotrichum orbiculare*.

Treatment	Conidial germination (%)	Appressorial formation (%)
SL06^a^	56.5 ± 1.2 B^b^	54.3 ± 1.6 b
SMG	100.0 ± 0.0 A	91.8 ± 3.0 a
sdH_2_O	100.0 ± 0.0 A	93.3 ± 2.6 a

*^a^SL06: Two hundred-fold diluted culture filtrate of S. griseorubiginosus LJS06; SMG, two hundred-fold diluted soybean meal broth; sdH_2_O: sterile distilled water.*

*^b^Means (n = 4) (± standard error) in the same column followed by different letters are significantly different (p < 0.05) according to Fisher’s least significant difference test.*

### LJS06 Culture Filtrate Reduced Anthracnose Severity in Cucumber Plants

**FIGURE 7:**
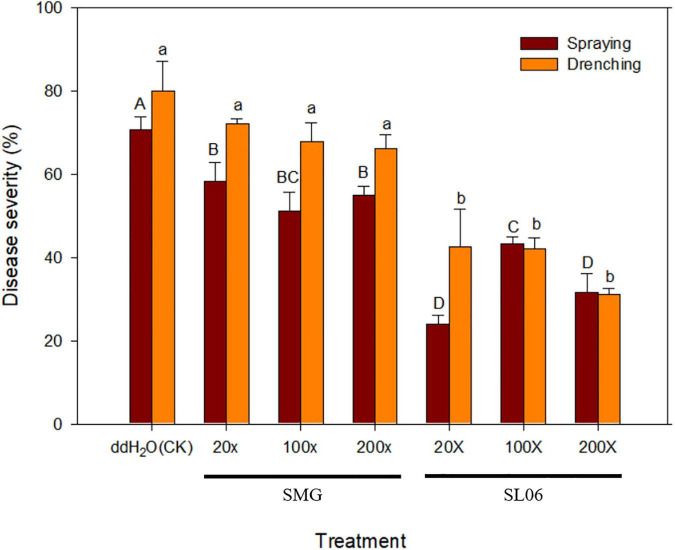
Effect of different dilutions of *Streptomyces griseorubiginosus* LJS06 culture filtrate (SL06) on cucumber anthracnose severity. Diluted SL06 solutions were sprayed or drenched 1 day before inoculation. Means (*n* = 6) for the same application method followed by different letters significantly differ (*p* < 0.05) according to Fisher’s least significant difference test. Bar = standard error.

To investigate the application method and dilution times of SL06 on managing cucumber anthracnose, two application methods (foliar spraying and soil drenching) with three dilution times (20×, 100×, and 200×) were included. After 11 days of inoculation, all dilutions of SL06 significantly (*p* < 0.05) reduced the anthracnose severity compared with water and diluted SMG broth treatments, regardless of the method used for applying the culture filtrate. The lowest disease severity was observed in foliar spraying and soil drenching of 20× dilution (24.2 and 20.5%, respectively), followed by 200× dilution (31.7 and 34.2%, respectively) and 100× dilution (43.3 and 41.7%, respectively) (Figure 7). The disease reduction effect of diluted SL06 was consistent with the results when the cucumber plants were only drenched with LJS06 spore suspension, suggesting induced resistance in cucumber by LJS06 and its culture filtrate.

## Discussion

Fungicides remain the most widely adopted approach of crop disease management in many countries. However, an overreliance on fungicides has raised many concerns pertaining to, for example, environmental pollution, fungicide residue, food safety, and the emergence of fungicide-resistant pathogens. This has necessitated the identification of fungicide alternatives while maintaining productivity and sustainability (Shaikh and Sayyed, 2015; Olanrewaju and Babalola, 2019).

In this study, we screened for bacteria with antagonistic and plant growth promotion–related traits. The efficacy of the bacterial strains as fungicide alternatives for managing cucumber anthracnose was also evaluated. Among the 28 bacteria obtained from the environment and different agricultural byproducts, four *Streptomyces* strains exhibited good *in vitro* antifungal activity. Of these, LJS06 was found to have good antifungal capability, promote plant growth significantly, and produce multiple secondary metabolites. In addition, drenching LJS06 spore suspension before inoculation significantly reduced anthracnose severity, suggesting multiple modes of action of LJS06 against anthracnose. On the basis of biochemical and morphological analyses, 16S rRNA sequences, and MLSA, LJS06 was identified to be *S. griseorubiginosus* (Ryabova and Preobrazhenskaya) Pridham et al.

Many antagonistic microorganisms produce various secondary metabolites. Some compounds in the metabolites exhibit excellent *in vitro* inhibition properties against fungal spore germination or mycelial growth (Köhl et al., 2019). In the present study, we observed that the mycelial growth of *C*. *orbiculare* was limited by *S. griseorubiginosus* LJS06 *in vitro*. Conidial germination and appressorium formation were also inhibited after treatment with diluted SL06, indicating a direct inhibition of the pathogen by the metabolites. Although the composition of the secondary metabolites produced by LJS06 was not further investigated, different strains of the same species have been reported to produce antibiotics, such as biphenomycins, cinerubins, reductiomycin, transcinnamamide, and tetraene KM-A, among which reductiomycin acts against Gram-positive bacteria, fungi, and Newcastle disease virus (David et al., 1980; Shimizu and Tamura, 1981; Ko and Bae, 1982; Ko et al., 1982; Ezaki et al., 1985, 1993).

In our results, the SL06-treated conidia of *C*. *orbiculare* turned blue after staining with Evans blue, suggesting that the metabolites in diluted SL06 may have altered the cell membrane permeability. Furthermore, SL06-treated conidia can oxidize DHR 123 into green fluorescent Rhodamine 123, suggesting that increased oxidative stress may be due to ROS accumulation in the conidia. Consequently, the energy metabolism and growth of *C*. *orbiculare* were inhibited. ROS play an essential role in fungal signaling and in regulating fungal development, particularly the formation of pathogenesis-associated structures (Heller and Tudzynski, 2011; Marschall and Tudzynski, 2016; Zhang et al., 2020). Therefore, imbalanced ROS accumulation in fungal cells may impair the mitochondrial or cell membrane, energy metabolism, or the differentiation of pathogenesis-associated structures (Ghezzi and Zeviani, 2012; Guo et al., 2013; Marschall and Tudzynski, 2016; Wang et al., 2017), which is consistent with our observation that conidial germination, respiration, and appressorium formation were significantly inhibited after SL06 treatment. In addition to direct inhibition of *C*. *orbiculare*, a reduction of anthracnose severity was observed when SL06 was drenched in soil before inoculation, suggesting that induced resistance may also be involved in cucumber plants against anthracnose. Because some metabolites produced by *Streptomyces* species, namely polyamines and hydrogen sulfide, may be involved in plant defense pathways (Walters, 2003; Choudhary et al., 2021), the functional metabolites in SL06 warrant further investigation.

Most streptomycetes are free-living in the soil as saprophytes, which can be recruited by root exudates and colonize the rhizosphere (Pieterse et al., 2014; Vurukonda et al., 2018). Suárez-Moreno et al. (2019) reported that *Streptomyces* sp. A20 can colonize the root hairs of two rice cultivars, which was hypothesized to be associated with the growth-promoting trait of the strain (Suárez-Moreno et al., 2019). In addition to epiphytic colonization on the rhizosphere, plant growth-promoting rhizobacteria were also documented entering the root tissues, mainly through wounds or cracks in the newly emerged roots, and survive as endophytes (Pieterse et al., 2014; Vurukonda et al., 2018). Some plant growth-promoting rhizobacteria can further penetrate plant tissues by producing extracellular enzymes, such as cellulase, chitinase, and pectinase (Hallmann et al., 1997; Compant et al., 2005; Vurukonda et al., 2018). Once colonized on the rhizosphere or entered plant tissues, these beneficial bacteria can trigger downstream signaling pathways associated with plant growth promotion or nutrition uptake or prime the plant host for disease resistance (Pieterse et al., 2014; Vurukonda et al., 2018).

In our screening and disease control experiments, the spore suspension and the cultural filtrate load are similar with previous studies (Patel et al., 2018; Fan et al., 2019). Drenching diluted culture filtrate of *S. griseorubiginosus* LJS06 was found to significantly reduce the anthracnose severity of cucumber plants, consistent with the result of our screening experiment. The spatial separation of LJS06, its metabolites, and *C*. *orbiculare* implies that LJS06 can prime cucumber plants for resistance through living cells or its metabolites. We also observed that LJS06 can produce several enzymes associated with active colonization or penetration of the host tissues, namely cellulase, chitinase, and protease, suggesting that LJS06 may also possess the capability to colonize or enter the root tissues. Future studies should investigate the rhizosphere capability of LJS06 and whether it can colonize in the host as an endophyte.

In conclusion, our findings revealed that LJS06 promotes plant growth and has multiple modes of action against cucumber anthracnose, which may be worth developing as a fungicide alternative for disease management.

## Data Availability Statement

The datasets presented in this study can be found in online repositories. The names of the repository/repositories and accession number(s) can be found below: https://www.ncbi.nlm.nih.gov/genbank/, OM877463; https://www.ncbi.nlm.nih.gov/genbank/, OM877464; https://www.ncbi.nlm.nih.gov/genbank/, OK668212; https://www.ncbi.nlm.nih.gov/genbank/, OL322103; https://www.ncbi.nlm.nih.gov/genbank/, OL322104; https://www.ncbi.nlm.nih.gov/genbank/, OL322105; and https://www.ncbi.nlm.nih.gov/genbank/, OM877465.

## Author Contributions

CHC and J-WH conceptualized and designed the study. CHC performed the experiments, collected the data, and wrote the first draft of the manuscript. C-FH organized and finalized the manuscript. J-WH and C-FH did funding acquisition. All authors contributed to the manuscript revision and read and approved the submitted version.

## Conflict of Interest

The authors declare that the research was conducted in the absence of any commercial or financial relationships that could be construed as a potential conflict of interest.

## Publisher’s Note

All claims expressed in this article are solely those of the authors and do not necessarily represent those of their affiliated organizations, or those of the publisher, the editors and the reviewers. Any product that may be evaluated in this article, or claim that may be made by its manufacturer, is not guaranteed or endorsed by the publisher.

## References

[B1] Abbasi S. Safaie N. Sadeghi A. Shamsbakhsh M. (2019). *Streptomyces* strains induce resistance to *Fusarium oxysporum* f. sp. *lycopersici* race 3 in tomato through different molecular mechanisms. *Front. Microbiol.* 10:1505. 10.3389/fmicb.2019.01505 31333615 PMC6616268

[B2] Baig U. Dahanukar N. Shintre N. Holkar K. Pund A. Lele U. (2021). Phylogenetic diversity and activity screening of cultivable actinobacteria isolated from marine sponges and associated environments from the western coast of India. *Access Microbiol.* 3:000242. 10.1099/acmi.0.000242 34712902 PMC8549387

[B3] Centore P. (2016). *SRGB Centroids for the ISCC-NBS Colour System 2 Colour Specifications.* London: Munsell Colour Science for Painters, 21.

[B4] Chen C. Dickman M. B. (2005). Proline suppresses apoptosis in the fungal pathogen *Colletotrichum trifolii*. *Proc. Natl. Acad. Sci.* 102 3459–3464. 10.1073/pnas.0407960102 15699356 PMC552905

[B5] Choudhary A. Singh S. Khatri N. Gupta R. (2021). Hydrogen sulphide: an emerging regulator of plant defence signalling. *Plant Biol.* [Online ahead of print]. 10.1111/plb.13376 34904345

[B6] Compant S. Reiter B. Sessitsch A. Nowak J. Clément C. Barka E. D. (2005). Endophytic colonization of *Vitis vinifera* L. by plant growth-promoting bacterium *Burkholderia* sp. strain PsJN. *Appl. Environ. Microbiol.* 71 1685–1693. 10.1128/AEM.71.4.1685-1693.2005 15811990 PMC1082517

[B7] Council of Agriculture, Executive Yuan (2020). *2020 Annual Report of Agricultural Statistics of Taiwan.* Taipei: Council of Agriculture, Executive Yuan, 340.

[B8] Cox K. D. Quello K. Deford R. J. Beckerman J. L. (2009). A rapid method to quantify fungicide sensitivity in the brown rot pathogen *Monilinia fructicola*. *Plant Dis.* 93 328–331. 10.1094/PDIS-93-4-0328 30764225

[B9] David L. Duteurtre M. Kergomard A. Kergomard G. Scanzi E. Staron T. (1980). Production of cinerubins by a *Streptomyces griseorubiginosus* strain. *J. Antibiotics* 33 49–53. 10.7164/antibiotics.33.49 7372549

[B10] Duncan S. M. Farrell R. L. Thwaites J. M. Held B. W. Arenz B. E. Jurgens J. A. (2006). Endoglucanase-producing fungi isolated from Cape Evans historic expedition hut on Ross Island, Antarctica. *Environ. Microbiol.* 8 1212–1219. 10.1111/j.1462-2920.2006.01013.x 16817929

[B11] El-Tarabily K. Nassar A. Hardy G. S. J. Sivasithamparam K. (2009). Plant growth promotion and biological control of *Pythium aphanidermatum*, a pathogen of cucumber, by endophytic actinomycetes. *J. Appl. Microbiol.* 106 13–26. 10.1111/j.1365-2672.2008.03926.x 19120624

[B12] Ezaki M. Iwami M. Yamashita M. Hashimoto S. Komori T. Umehara K. (1985). Biphenomycins A and B, novel peptide antibiotics I. Taxonomy, fermentation, isolation and characterization. *J. Antibiot.* 38 1453–1461. 10.7164/antibiotics.38.1453 3841122

[B13] Ezaki M. Shigematsu N. Yamashita M. Komori T. Umehara K. Imanaka H. (1993). Biphenomycin C, a precursor of biphenomycin A in mixed culture. *J. Antibiot.* 46 135–140. 10.7164/antibiotics.46.135 8436546

[B14] Fan Y. T. Chung K. R. Huang J. W. (2019). Fungichromin production by *Streptomyces padanus* PMS-702 for controlling cucumber downy mildew. *Plant Pathol. J.* 35 341–350. 10.5423/PPJ.OA.03.2019.0057 31481857 PMC6706012

[B15] Gardes M. Bruns T. D. (1993). ITS primers with enhanced specificity for basidiomycetes-application to the identification of mycorrhizae and rusts. *Mol. Ecol.* 2 113–118. 10.1111/j.1365-294x.1993.tb00005.x 8180733

[B16] Ghezzi D. Zeviani M. (2012). Assembly factors of human mitochondrial respiratory chain complexes: physiology and pathophysiology. *Adv. Exp. Med. Biol.* 748 65–106. 10.1007/978-1-4614-3573-0_4 22729855

[B17] Glickmann E. Dessaux Y. (1995). A critical examination of the specificity of the salkowski reagent for indolic compounds produced by phytopathogenic bacteria. *Appl. Environ. Microbiol.* 61 793–796. 10.1128/aem.61.2.793-796.1995 16534942 PMC1388360

[B18] Guo C. Sun L. Chen X. Zhang D. (2013). Oxidative stress, mitochondrial damage and neurodegenerative diseases. *Neural Regen. Res.* 8 2003–2014. 25206509 10.3969/j.issn.1673-5374.2013.21.009 PMC4145906

[B19] Guo Y. Zheng W. Rong X. Huang Y. (2008). A multilocus phylogeny of the *Streptomyces griseus* 16S rRNA gene clade: use of multilocus sequence analysis for streptomycete systematics. *Int. J. Syst. Evol. Microbiol.* 58 149–159. 10.1099/ijs.0.65224-0 18175701

[B20] Halimahtussadiyah R. Natsir M. Kurniawati D. Utamy S. P. (2017). Isolation and identification of chitinolytic bacteria of pohara river of South East Sulawesi and the optimization production of chitinase enzyme. *Int. Conf. Chem. Chem. Proc. Eng.* 1823:020062.

[B21] Hallmann J. Quadt-Hallmann A. Mahaffee W. Kloepper J. (1997). Bacterial endophytes in agricultural crops. *Can. J. Microbiol.* 43 895–914. 10.1139/m97-131

[B22] Han J.-H. Cho M.-H. Kim S. B. (2012). Ribosomal and protein coding gene based multigene phylogeny on the family *Streptomycetaceae*. *Syst. Appl. Microbiol.* 35 1–6. 10.1016/j.syapm.2011.08.007 22154623

[B23] Hayakawa M. Honomura H. (1987). Humic acid-vitamin agar, a new medium for the selective isolation of soil actinomycetes. *J. Ferment. Technol.* 65 501–509. 10.1016/0385-6380(87)90108-7

[B24] Heller J. Tudzynski P. (2011). Reactive oxygen species in phytopathogenic fungi: signaling, development, and disease. *Annu. Rev. Phytopathol.* 49 369–390. 10.1146/annurev-phyto-072910-095355 21568704

[B25] Jankowitsch F. Schwarz J. Rückert C. Gust B. Szczepanowski R. Blom J. (2012). Genome sequence of the bacterium *Streptomyces davawensis* JCM 4913 and heterologous production of the unique antibiotic roseoflavin. *J. Bacteriol.* 194 6818–6827. 10.1128/JB.01592-12 23043000 PMC3510588

[B26] Jia H. Wang H. (2021). *Cucumber Economic Values and Its Cultivation and Breeding.* London: IntechOpen, 228.

[B27] Jiang H. Han L. Li J. Yu M. Zhao J. Guo X. (2020). *Streptomyces montanus* sp. nov., a novel actinomycete isolated from soil. *Int. J. Syst. Evol. Microbiol.* 70 3226–3233. 10.1099/ijsem.0.004160 32375929

[B28] Kannan K. Jain S. K. (2000). Oxidative stress and apoptosis. *Pathophysiology* 7 153–163. 10.1016/S0928-4680(00)00053-5 10996508

[B29] Kelly K. (1964). *Color-name Charts Illustrated with Centroid Colors.* Chicago: Inter-Society Color Council-National Bureau of Standards, 18.

[B30] Khokhar I. Mukhtar I. Mushtaq S. (2011). Comparative studies on the amylase and cellulase production of *Aspergillus* and *Penicillium*. *J. Appl. Sci. Environ. Manage.* 15 657–661.

[B31] Ko Y.-H. Bae M. (1982). Studies on the antifungal antibiotics produced by a *Streptomyces* sp. (Part 4) the occurrence of tetraene substance and its physiological properties. *Microbiol. Biotechnol. Lett.* 10 211–215.

[B32] Ko Y.-H. Jung S.-H. Bae M. (1982). Studies on the antifungal antibiotics produced by a *Streptomyces* sp.(Part 3) microbiological properties of the strain. *Microbiol. Biotechnol. Lett.* 10 117–122.

[B33] Köhl J. Kolnaar R. Ravensberg W. J. (2019). Mode of action of microbial biological control agents against plant diseases: relevance beyond efficacy. *Front. Plant Sci.* 10:845. 10.3389/fpls.2019.00845 31379891 PMC6658832

[B34] Kumar S. Stecher G. Li M. Knyaz C. Tamura K. (2018). MEGA X: molecular evolutionary genetics analysis across computing platforms. *Mol. Biol. Evol.* 35 1547–1549. 10.1093/molbev/msy096 29722887 PMC5967553

[B35] Kwack M. S. Kim E. N. Lee H. Kim J.-W. Chun S.-C. Kim K. D. (2005). Digital image analysis to measure lesion area of cucumber anthracnose by *Colletotrichum orbiculare*. *J. Gen. Plant Pathol.* 71 418–421. 10.1007/s10327-005-0233-0

[B36] Labeda D. P. Dunlap C. A. Rong X. Huang Y. Doroghazi J. R. Ju K.-S. (2017). Phylogenetic relationships in the family *Streptomycetaceae* using multi-locus sequence analysis. *Antonie Van Leeuwenhoek* 110 563–583. 10.1007/s10482-016-0824-0 28039547 PMC10327403

[B37] Labeda D. P. Goodfellow M. Brown R. Ward A. C. Lanoot B. Vanncanneyt M. (2012). Phylogenetic study of the species within the family *Streptomycetaceae*. *Antonie Van Leeuwenhoek* 101 73–104. 10.1007/s10482-011-9656-0 22045019

[B38] Lechevalier M. (1980). “The chemotaxonomy of actinomycetes,” in *Actinomycetes Taxonomy*, eds Dietz A. Thayer D. W. (Arlington, VA: Virginia Society of Industrial Microbiology), 227–291.

[B39] Lechuga E. G. O. Zapata I. Q. Niño K. A. (2016). Detection of extracellular enzymatic activity in microorganisms isolated from waste vegetable oil contaminated soil using plate methodologies. *Afr. J. Biotechnol.* 15 408–416. 10.5897/AJB2015.14991

[B40] Li K. Guo Y. Wang J. Wang Z. Zhao J. Gao J. (2020). *Streptomyces aquilus* sp. nov., a novel actinomycete isolated from a Chinese medicinal plant. *Int. J. Syst. Evol. Microbiol.* 70 1912–1917. 10.1099/ijsem.0.003995 31967952

[B41] Maleki H. Dehnad A. Hanifian S. Khani S. (2013). Isolation and molecular identification of *Streptomyces* spp. with antibacterial activity from northwest of Iran. *Bioimpacts* 3 129–134. 10.5681/bi.2013.017 24163805 PMC3786795

[B42] Marschall R. Tudzynski P. (2016). Reactive oxygen species in development and infection processes. *Semin. Cell Dev. Biol.* 57 138–146. 10.1016/j.semcdb.2016.03.020 27039026

[B43] Meera M. Shivanna M. Kageyama K. Hyakumachi M. (1994). Plant growth promoting fungi from zoysiagrass rhizosphere as potential inducers of systemic resistance in cucumbers. *Phytopathology* 84 1399–1406. 10.1094/Phyto-84-1399

[B44] Mehling A. Wehmeier U. F. Piepersberg W. (1995). Nucleotide sequences of streptomycete 16S ribosomal DNA: towards a specific identification system for streptomycetes using PCR. *Microbiology* 141 2139–2147. 10.1099/13500872-141-9-2139 7496525

[B45] Mun B.-G. Lee W.-H. Kang S.-M. Lee S.-U. Lee S.-M. Lee D. Y. (2020). *Streptomyces* sp. LH 4 promotes plant growth and resistance against *Sclerotinia sclerotiorum* in cucumber via modulation of enzymatic and defense pathways. *Plant Soil* 448 87–103. 10.1007/s11104-019-04411-4

[B46] Mundt J. O. Hinkle N. F. (1976). Bacteria within ovules and seeds. *Appl. Environ. Microbiol.* 32 694–698. 10.1128/aem.32.5.694-698.1976 984839 PMC170385

[B47] Nassar A. H. El-Tarabily K. A. Sivasithamparam K. (2003). Growth promotion of bean (*Phaseolus vulgaris* L.) by a polyamine-producing isolate of *Streptomyces griseoluteus*. *Plant Growth Regul.* 40 97–106. 10.1023/A:1024233303526

[B48] Nei M. Kumar S. (2000). *Molecular Evolution and Phylogenetics.* Oxford: Oxford University Press, 348.

[B49] Ntabo R. M. Nyamache A. K. Lwande W. Kabii J. Nonoh J. (2018). Enzymatic activity of endophytic bacterial isolates from selected mangrove plants in Kenya. *Open Microbiol. J.* 12 354–363. 10.2174/1874285801812010354

[B50] Olanrewaju O. S. Babalola O. O. (2019). *Streptomyces*: implications and interactions in plant growth promotion. *Appl. Microbiol. Biotechnol.* 103 1179–1188. 30594952 10.1007/s00253-018-09577-y PMC6394478

[B51] Palaniyandi S. Yang S. Suh J. W. (2013). Extracellular proteases from *Streptomyces phaeopurpureus* ExPro138 inhibit spore adhesion, germination and appressorium formation in *Colletotrichum coccodes*. *J. Appl. Microbiol.* 115 207–217. 10.1111/jam.12212 23560777

[B52] Paris H. S. Daunay M.-C. Janick J. (2012). Occidental diffusion of cucumber (*Cucumis sativus*) 500-1300 CE: two routes to Europe. *Ann. Bot.* 109 117–126. 10.1093/aob/mcr281 22104164 PMC3241595

[B53] Patel J. K. Madaan S. Archana G. (2018). Antibiotic producing endophytic *Streptomyces* spp. colonize above-ground plant parts and promote shoot growth in multiple healthy and pathogen-challenged cereal crops. *Microbiol. Res.* 215 36–45. 10.1016/j.micres.2018.06.003 30172307

[B54] Perwendha R. Oetari A. Sjamsuridzal W. (2020). Skimmed milk-degrading ability of *Rhizopus azygosporus* UICC 539 at various temperatures. *AIP Conf. Proc.* 2242 050006.

[B55] Pettit R. K. Weber C. A. Kean M. J. Hoffmann H. Pettit G. R. Tan R. (2005). Microplate Alamar blue assay for *Staphylococcus epidermidis* biofilm susceptibility testing. *Antimicrob. Agents Chemother.* 49 2612–2617. 10.1128/AAC.49.7.2612-2617.2005 15980327 PMC1168683

[B56] Pieterse C. M. Zamioudis C. Berendsen R. L. Weller D. M. Van Wees S. C. Bakker P. A. (2014). Induced systemic resistance by beneficial microbes. *Ann. Rev. Phytopathol.* 52 347–375. 24906124 10.1146/annurev-phyto-082712-102340

[B57] Rong X. Huang Y. (2010). Taxonomic evaluation of the *Streptomyces griseus* clade using multilocus sequence analysis and DNA–DNA hybridization, with proposal to combine 29 species and three subspecies as 11 genomic species. *Int. J. Syst. Evol. Microbiol.* 60 696–703. 10.1099/ijs.0.012419-0 19656940

[B58] Sardi P. Saracchi M. Quaroni S. Petrolini B. Borgonovi G. Merli S. (1992). Isolation of endophytic *Streptomyces* strains from surface-sterilized roots. *Appl. Environ. Microbiol.* 58 2691–2693. 16348757 10.1128/aem.58.8.2691-2693.1992 PMC195843

[B59] Schwyn B. Neilands J. (1987). Universal chemical assay for the detection and determination of siderophores. *Anal. Biochem.* 160 47–56. 10.1016/0003-2697(87)90612-9 2952030

[B60] Shaikh S. Sayyed R. (2015). “Role of plant growth-promoting rhizobacteria and their formulation in biocontrol of plant diseases,” in *Plant Microbes Symbiosis: Applied Facets*, ed. Arora N. K. (New Delhi: Springer), 337–351. 10.1007/s00203-021-02492-3

[B61] Sharma N. Arya G. Kumari R. M. Gupta N. Nimesh S. (2019). Evaluation of anticancer activity of silver nanoparticles on the A549 human lung carcinoma cell lines through alamar blue assay. *Bio-Protocol* 9:e3131. 10.21769/BioProtoc.3131 33654760 PMC7854262

[B62] Shih H. D. Liu Y.-C. Hsu F. L. Mulabagal V. Dodda R. Huang J. W. (2003). Fungichromin : a substance from *Streptomyces padanus* with inhibitory effects on *Rhizoctonia solani*. *J. Agric. Food Chem.* 51 95–99. 10.1021/jf025879b 12502391

[B63] Shimizu K. Hossain M. M. Kato K. Kubota M. Hyakumachi M. (2013). Induction of defense responses in cucumber plants by using the cell-free filtrate of the plant growth-promoting fungus *Penicillium simplicissimum* GP17-2. *J. Oleo Sci.* 62 613–621. 10.5650/jos.62.613 23985491

[B64] Shimizu K. Tamura G. (1981). Reductiomycin, a new antibiotic. I. Taxonomy, fermentation, isolation, characterization and biological activities. *J. Antibiot.* 34 649–653. 10.7164/antibiotics.34.649 7275849

[B65] Shirling E. T. Gottlieb D. (1966). Methods for characterization of *Streptomyces* species. *Int. J. Syst. Bacteriol.* 16 313–340.

[B66] Shoresh M. Yedidia I. Chet I. (2005). Involvement of jasmonic acid/ethylene signaling pathway in the systemic resistance induced in cucumber by *Trichoderma asperellum* T203. *Phytopathology* 95 76–84. 10.1094/PHYTO-95-0076 18943839

[B67] Sturz A. Nowak J. (2000). Endophytic communities of rhizobacteria and the strategies required to create yield enhancing associations with crops. *Appl. Soil Ecol.* 15 183–190.

[B68] Suárez-Moreno Z. R. Vinchira-Villarraga D. M. Vergara-Morales D. I. Castellanos L. Ramos F. A. Guarnaccia C. (2019). Plant-growth promotion and biocontrol properties of three *Streptomyces* spp. isolates to control bacterial rice pathogens. *Front. Microbiol.* 10:290. 10.3389/fmicb.2019.00290 30858835 PMC6398372

[B69] Tang Q. Bie X. Lu Z. Lv F. Tao Y. Qu X. (2014). Effects of fengycin from *Bacillus subtilis* fmbJ on apoptosis and necrosis in *Rhizopus stolonifer*. *J. Microbiol.* 52 675–680. 10.1007/s12275-014-3605-3 25098563

[B70] Vurukonda S. S. K. P. Giovanardi D. Stefani E. (2018). Plant growth promoting and biocontrol activity of *Streptomyces* spp. as endophytes. *Int. J. Mol. Sci.* 19 952–977. 10.3390/ijms19040952 29565834 PMC5979581

[B71] Walters D. (2003). Resistance to plant pathogens: possible roles for free polyamines and polyamine catabolism. *New Phytol.* 159 109–115. 10.1046/j.1469-8137.2003.00802.x 33873679

[B72] Wang T.-Y. Libardo M. D. J. Angeles-Boza A. M. Pellois J.-P. (2017). Membrane oxidation in cell delivery and cell killing applications. *ACS Chem. Biol.* 12 1170–1182. 10.1021/acschembio.7b00237 28355059 PMC5905413

[B73] White T. J. Bruns T. Lee S. Taylor J. (1990). “Amplification and direct sequencing of fungal ribosomal RNA genes for phylogenetics,” in *PCR Protocols: a Guide to Methods Applications*, eds Michael A. I. David H. G. John J. S. Thomas J. W. (San Diego, CA: Harcourt Brace Jovanovich), 315–322. 10.1016/B978-0-12-372180-8.50042-1

[B74] Yang C.-J. Huang T.-P. Huang J.-W. (2021). Field sanitation and foliar application of *Streptomyces padanus* PMS-702 for the control of rice sheath blight. *Plant Pathol. J.* 37 57–71. 10.5423/PPJ.OA.12.2020.0227 33551697 PMC7847755

[B75] Zhang Z. Chen Y. Li B. Chen T. Tian S. (2020). Reactive oxygen species: a generalist in regulating development and pathogenicity of phytopathogenic fungi. *Comput. Struct. Biotechnol. J.* 18 3344–3349. 10.1016/j.csbj.2020.10.024 33294130 PMC7677654

