# Supplementary figures and images for: Identification and Characterization of a Multifunctional Biocontrol Agent, Streptomyces griseorubiginosus LJS06, Against Cucumber Anthracnose
Source: Front Microbiol. 2022 Jun 2;13:923276. doi: 10.3389/fmicb.2022.923276 (PMC9201727; doi:10.3389/fmicb.2022.923276)

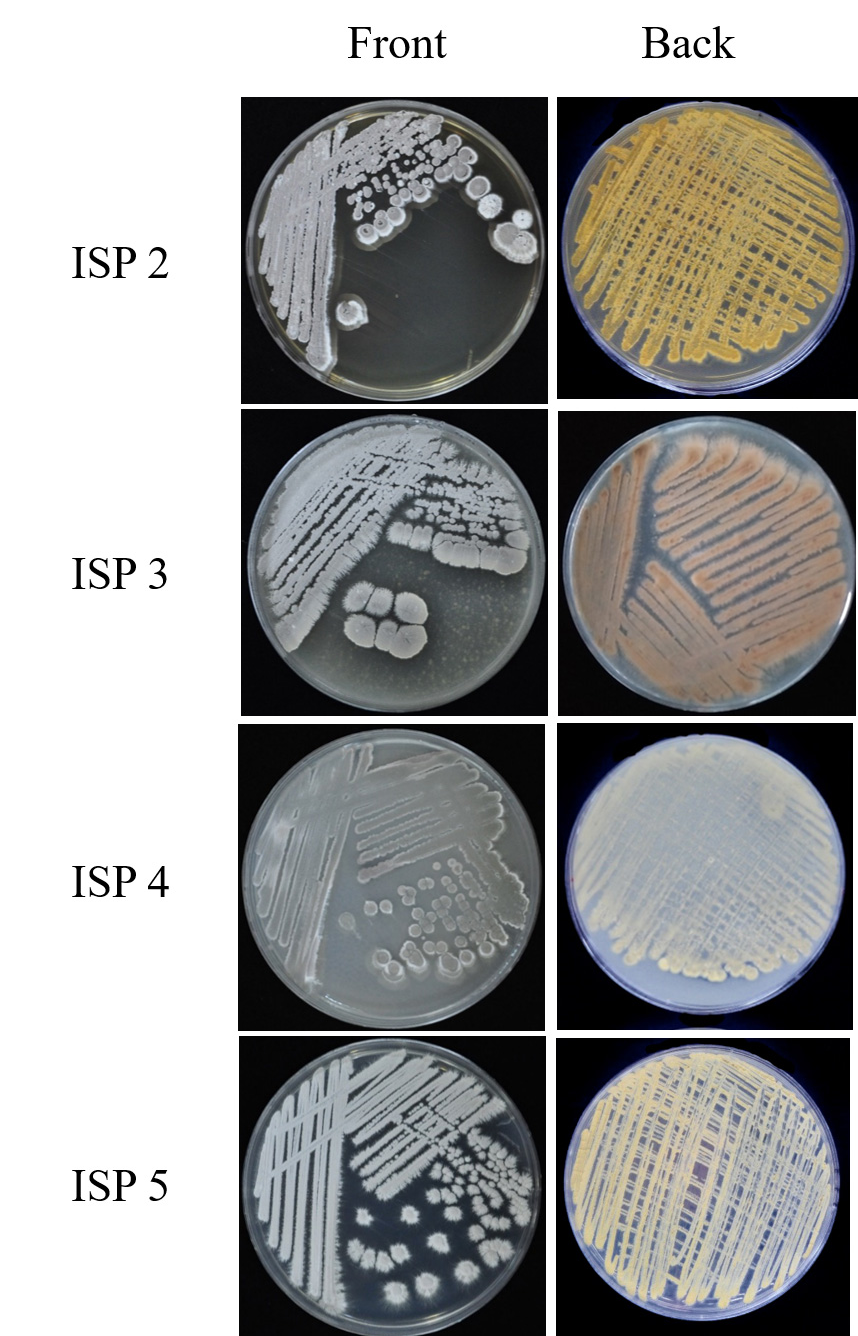

Supplement: Supplementary file 1 [file Image_1.TIF]

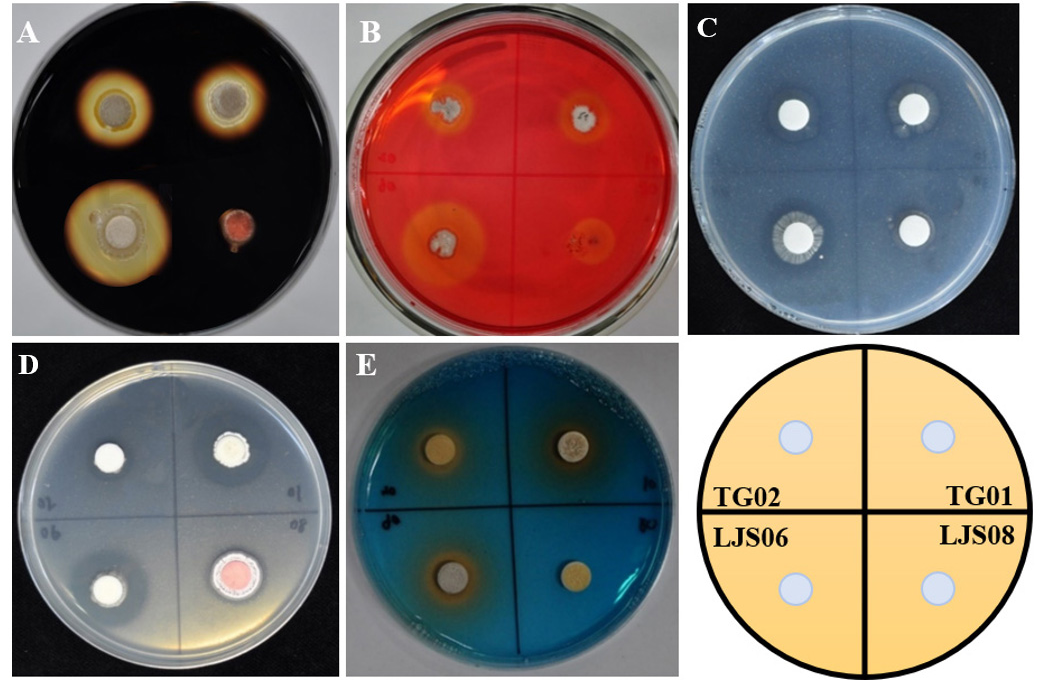

Supplement: Supplementary file 2 [file Image_2.TIF]

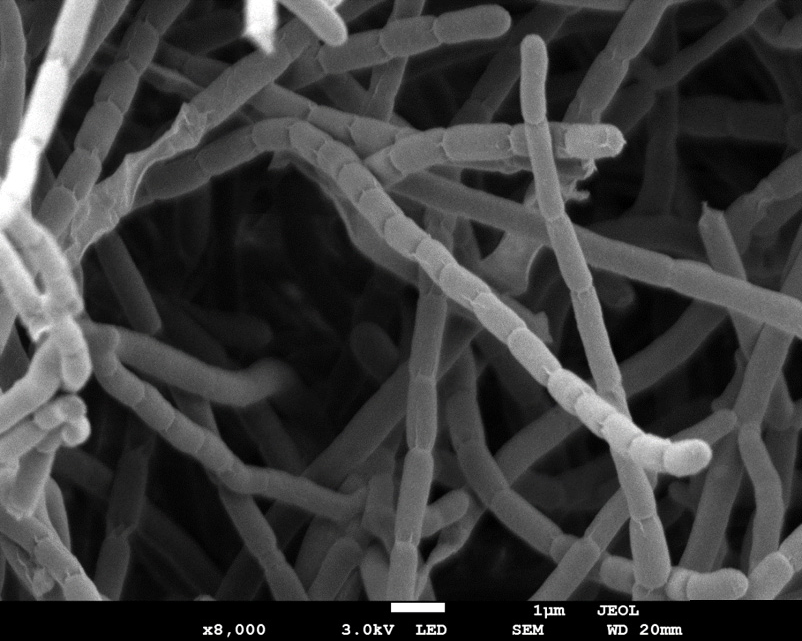

Supplement: Supplementary file 3 [file Image_3.TIF]
