# Supplementary material for: Identification and Characterization of a Multifunctional Biocontrol Agent, Streptomyces griseorubiginosus LJS06, Against Cucumber Anthracnose
Source: Front Microbiol. 2022 Jun 2;13:923276. doi: 10.3389/fmicb.2022.923276 (PMC9201727; doi:10.3389/fmicb.2022.923276)
Supplement: Supplementary file 4 [file Table_1.pdf]

Supplementary Table 1. Primers used for PCR amplification in this study

Gene	Primers	Primer sequence (5'-3')	References
ITS	ITS1-F	CTTGGTCATTTAGAGGAAGTAC	White et al. (1990);
	ITS4	TCCTCCGCTTATTGATATGC	Gardes and Bruns (1993)
16S rRNA	St-F	AAGCCCTGGAAACGGGGT	Maleki et al. (2013)
	St-R	CGTGTGCAGCCCAAGACA	
atpD	atpDPF	GTCGGCGACTTCACCAAGGGCAAG GTGTTCAACACC	Guo et al. (2008)
	atpDPR	GTGAACTGCTTGGCGACGTGGGTGTTCTGGGACAGGAA	
rpoB	rpoBPF	GAGCGCATGACCACCCAGGACGTCGAGGC	Guo et al. (2008)
	rpoBPR	CCTCGTAGTTGTGACCCTCCCACGGCATGA	
trpB	trpBPF	GCGCGAGGACCTGAACCACACCGGCTCACACAAGATCAACA	Guo et al. (2008)
	trpBPR	TCGATGGCCGGGATGATGCCCTCGGTGCGCGACAGCAGGC	
