# Supplementary material for: Identification and Characterization of a Multifunctional Biocontrol Agent, Streptomyces griseorubiginosus LJS06, Against Cucumber Anthracnose
Source: Front Microbiol. 2022 Jun 2;13:923276. doi: 10.3389/fmicb.2022.923276 (PMC9201727; doi:10.3389/fmicb.2022.923276)
Supplement: Supplementary file 5 [file Table_2.pdf]

Supplementary Table 2. NCBI GenBank accession numbers of the gene sequences for the reference strains and the *Streptomyces* strains used in this study

Species	Strain	GeneBank Accession numbers				References
		16S rRNA	*atpD*	*rpoB*	*trpB*	
Streptomyces sp.	TG01	OM877463	-	-	-	This study
Streptomyces sp.	TG02	OM877464	-	-	-	This study
Streptomyces griseorubiginosus	LJS06	OK668212	OL322103	OL322104	OL322105	This study
Streptomyces asterosporus	LJS08	OM877465	-	-	-	This study
Streptomyces sp.	MGB 2771	MN339842	-	-	-	Baig et al. (2021)
Streptomyces antibioticus	NRRL B-1701	AY999776	KT384466	KT388785	KT389135	Labeda et al. (2012, 2017)
Streptomyces aquilus	GGCR-6	MH718844	AZP20447	AZP19751	AZP17092	Li et al. (2020)
Streptomyces asterosporus	NRRL B-24328	AY999902	KT384471	KT388790	KT389140	Labeda et al. (2012, 2017)
Streptomyces chartreusis	ISP 5085	-	KT384507	KT388826	KT389176	Labeda et al. (2017)
Streptomyces ciscaucasicus	DSM 40275	AY508512	-	-	-	Kämpfer et al. (2018)
	KCTC 19958	-	JF424194	JF424007	JF423960	Han et al. (2012)
Streptomyces coacervatus	IFM 11055	NR112916	-	-	-	Shibazaki et al. (2011)
	DSM 41983	-	MN478476	MN478477	MN478478	Jiang et al. (2020)
Streptomyces flavidovirens	NBRC 13039	AB184270	-	-	-	Labeda et al. (2012)
	KCTC 19960	-	JF424196	JF424009	JF423962	Han et al. (2012)
Streptomyces griseorubiginosus	AS 4.1766	FJ405905	EF661718	EF661781	EF661802	Rong & Huang (2010)
Streptomyces lincolnensis	NRRL 2936	NR119101	-	-	-	Mehling et al. (1995)
	ISP 5355	-	KT384623	KT388943	KT389292	Labeda et al. (2017)
Streptomyces phaepurpureus	NRRL B-2260	DQ026666	KT384682	KT389003	KT389352	Labeda et al. (2017)
Kitasatospora azatica	NBRC 13803	NR112415	-	-	-	Labeda et al. (2012)
	KCTC 9699	-	JF424212	JF424025	JF423978	Han et al. (2012)
